# Strengthening care for children with complex mental health conditions: Views of Australian clinicians

**DOI:** 10.1371/journal.pone.0214821

**Published:** 2019-04-02

**Authors:** Kate Paton, Harriet Hiscock

**Affiliations:** 1 Health Services, Centre for Community Child Health, Murdoch Children’s Research Institute, Melbourne, Victoria, Australia; 2 Health Services Unit, The Royal Children’s Hospital, Melbourne, Australia; 3 Department of Paediatrics, The University of Melbourne, Melbourne, Australia; National University of Ireland Galway, IRELAND

## Abstract

**Objectives:**

Improving mental health outcomes for children and young people has become a priority for policy makers in the developed world. In Australia, up to half of all children and adolescents meeting criteria for mental health disorders receive suboptimal levels of treatment (or no treatment at all) despite the availability of effective treatments. Children with complex mental health conditions are particularly at risk of inadequate treatment as optimal care requires coordination from medical, educational and social services. In Australia, clinicians including pediatricians, psychologists and child and adolescent psychiatrists deliver the bulk of mental health care for children with complex mental health conditions. We aimed to determine perspectives of these Australian clinicians on barriers and enablers within the current system and components of an optimal model of care.

**Methods:**

Inductive content analysis was used to analyse 30 semi-structured interviews with key clinicians managing the care of children with complex mental health conditions across Australia. Interviews were conducted using vignettes with Attention Deficit Hyperactivity Disorder (ADHD) and Autism as exemplars.

**Findings:**

Multiple barriers to optimal care exist at a systemic, clinician and family level. However, regional health systems provide an enabling environment from which metropolitan models could learn. Transitioning to adult services was highlighted as the most compromised area of care. Clinicians identified short (e.g. empowering parents to advocate for and deliver their child’s care, case conferencing with schools) and long term (e.g. co-locating disciplines to deliver care, workforce training) solutions.

**Conclusions:**

Whilst multiple barriers to optimal care for children with complex mental health conditions exist, clinicians identify several enablers including developing networks with other disciplines and empowering parents to advocate for and co-ordinate care. Systemic changes based on multidisciplinary, co-located and integrated care services should be developed as longer term solutions.

## Introduction

Mental health conditions have long been identified as a leading cause of disability in young people worldwide. [1, 2] Improving access to mental health care for young people has become a focus for policy makers internationally. Governments in England and Scotland have recently undertaken major reviews of preventative strategies and service provision for children and adolescents with mental health conditions. [3, 4] Multiple organisations in the United States have identified challenges with having the capacity to provide evidence based mental health services, in particular the lack of capacity within the mental health workforce for children and adolescents. [5, 6, 7]

Consistent with prevalence figures in other developed countries [3, 4] in Australia 14% of 4–17 year olds or 580,000 children and adolescents meet diagnostic criteria for at least one mental health disorder over a 12 month period. [8] Comorbid conditions are common, creating additional complexity. Left unmanaged, children’s development, education and opportunity to fulfil their potential is at risk [9] and the cost to community substantial [10] with 50% of mental health problems arising in childhood persisting into adulthood. [11] However, despite the availability of effective treatments, around half of all Australian children and adolescents meeting criteria for mental health conditions receive sub-optimal management or do not access treatment at all. [8] Previous Australian studies have identified access may be impacted by parental perceptions, cost, transport, knowing where to go, stigma and community attitudes to mental health. [12,13]. In addition, rural communities identify a lack of local qualified child and adolescent professionals, social gossiping and high visibility (linked to stigma) [14, 15]. Conversely, facilitators include mental health literacy and past positive experiences [12].

Complex care needs are those resulting from one or more conditions which require access to multiple health and social support services. [16] Conditions such as Attention Deficit Hyperactivity Disorder (ADHD) and Autism are examples of conditions [17] with complex care needs. In Australia, ADHD is the most common mental health condition amongst 4–17 year olds affecting 7.5% [8] whilst Autism is estimated to affect between 1.5–2.5% of children. [18]

Delivery of services within the Australian healthcare system is complex in part due to the different roles and responsibilities of various arms of government. [19, 20] For example, the Commonwealth government funds community-based primary (e.g. general practitioners) and secondary healthcare clinicians (e.g. pediatricians, psychologists and child and adolescent psychiatrists) via the Medicare Benefits Scheme (MBS), a universal insurance scheme. General Practitioners act as gatekeepers to secondary healthcare clinicians who require a referral from a General Practitioner in order for the family to receive a MBS rebate for their child’s consultation with the secondary healthcare clinician. State and territory governments fund Child and Adolescent Mental Health Services (CAMHS). Further, many clinicians working in private practice in the community charge patients over and above MBS fees, leading to patient out-of-pocket costs for each episode of care. In addition, in terms of social and community-based care, Australia’s recently introduced National Disability Insurance Scheme (NDIS) is an independent statutory authority charged with providing long term support to those with a significant and permanent disability.

Worldwide, delivery of coordinated care to children with complex needs is often overlooked, even though children, by virtue of their youth, stand to benefit more than adults from coordinated care in terms of quality of life years gained. [16] The need for further research into system level intersectoral linkages between mental health and nonclinical sectors specifically for children and adolescents was highlighted in a recent systematic review. [21] Clinicians including pediatricians, psychologists and child and adolescent psychiatrists deliver the bulk of mental health care for Australian children with complex mental health conditions. [8] Despite this, no studies have utilised their expertise in critiquing systems of care for these children, thereby ignoring a valuable source of feedback and potential solutions for optimal care.

We therefore aimed to determine perspectives of Australian pediatricians, psychologists, and child and adolescent psychiatrists on:

1. the barriers to access and optimal care within the current health system for children with complex mental health conditions;
2. enablers to access and optimal care; and
3. components of an optimal model of care.

## Method

This study was nested within a mixed methods study known as Models of Child Health Appraised (MOCHA). [17] MOCHA is a multi-country study which systematically reviewed variation in health systems for children in Europe with the aim of identifying optimal models of care. Vignettes [22, 23, 24] were developed for children with complex mental health conditions, using ADHD and Autism as exemplars. [17] The qualitative methodology for this project was based on interpretative description, a methodology frequently used in healthcare research. [25] Interpretative description is an applied qualitative methodology which aims to generate an understanding of complex clinical phenomena which can then lead to applied outcomes in real world circumstances. [26]

### Recruitment

A purposive sampling strategy using the professional networks of the principal investigator Harriet Hiscock (pediatrician) was used initially. Key informants [27] were identified from within professional networks of pediatricians, child and adolescent psychiatrists and psychologists. Recruitment was supplemented by snowball sampling [28] by asking participants involved in the study to identify other people who may be able to comment on current services for children with ADHD and Autism. [29] To ensure a multi-disciplinary, quasi-national view of service delivery across Australia, we interviewed healthcare professionals in metropolitan and rural locations, in all Australian States and territories, (except the Australian Capital Territory because none of the clinicians approached responded).

### Procedures

KP conducted semi-structured interviews with 30 health professionals (13 pediatricians, 10 child and adolescent psychiatrists, 6 psychologists and 1 health system professional from another background) from July 2016 to March 2017. Approximately half the participants were female (16/30–53%). One third (10/30) worked in rural or regional settings including 10% of participants (3/30) who worked in remote areas of Australia with a large indigenous population. Almost half of the participants (13/30–43%) were engaged in both public and privately funded health care settings with others working exclusively in either public or private settings. There was a range of experience amongst participants from a trainee psychiatrist and junior consultant to experienced professionals nearing retirement in their respective professions although overall the participant group were more experienced health care professionals. Approval was received from Human Research Ethics Committee at The Royal Children’s Hospital, Melbourne, Victoria (HREC 36217) prior to commencement of the project. Prior to interviews participants were informed that their participation was voluntary and unpaid and verbal informed consent was sought.

Participants were invited to address questions from the MOCHA surveys [17], relating to Autism only, (8 participants) ADHD only (3 participants) or both Autism and ADHD (19 participants), according to their clinical casemix (S1, S2 and S3 Files). As the project progressed, interview questions were adapted following participant responses. Interviews lasted approximately one hour with participants interviewed either in person or by phone. Detailed field notes were kept for all interviews. Interviews were audio-recorded (with consent), de-identified and transcribed. Transcripts were cross-referenced with the recordings to ensure accuracy and then coded for analysis using NVIVO 11.0 [30] a software programme that allows for coding and theming of qualitative data.

### Analyses

The broad study aims provided the broad focus for analysis but following coding more "interpretive" sub-themes were developed using an inductive approach. [31] The objective of the analysis was to identify constructs, i.e. provisional inferences drawn from statements and observations. By using the cross-referencing ability of the NVIVO software, statements relevant to each construct were reviewed. Categories were developed in line with the primary research questions and themes were then identified using the process of content analysis. [31] The analyst was a female Master of Public Health (KP) not from a healthcare background. KP maintained a reflexivity journal throughout the interview and analysis process. A coding schema was developed by KP and the principal investigator (HH) and applied to all transcripts. The transcripts were co-read by a Master of Public Health student (VW) and themes discussed with the research group including the principal investigator (HH) to achieve consensus. The final summary of key themes was forwarded to participants (pseudonymised quotes were included) for feedback and comment, recognising that the validity of member checking is debated in the literature. [32]. Findings are reported in line with the COnsolidated criteria for REporting Qualitative research (COREQ) [33] checklist.

## Results

Analysis of the interview transcripts revealed important themes which are presented using representative quotes. Quotes have been truncated where necessary without changing the meaning. This is represented by an ellipsis.

### Aim 1. Barriers to access and optimal care

#### Healthcare system factors

Participants identified few national policies or procedures operating across Australia for coordination of care for children with complex mental health conditions. The socio-political structure of the healthcare system in Australia (i.e. responsibility shared between the Commonwealth and State governments) and the pluralistic nature of service delivery with both publicly funded and private fee-for-service providers adds complexity to service provision.

“*I think that there is an opportunity for better coordination across the various arms of care provision*. *You gonna have the NDIS system*, *and you've got Medicare system*, *and the state health system*. *And I think it would be good to make sure that these have good interfaces so as to minimise*, *if you like*, *people falling through the gaps”*. *(Child and Adolescent Psychiatrist 9)*

“*the fact that Australia has much more pluralistic healthcare system…*..*than may occur in some other countries*, *that's the downside*. *It has many upsides of course but the downsides are that it's much more variable*, *and it's harder to come up with coordinated plans*.*” (Child and Adolescent Psychiatrist 9)*

Wide variation in responses between participants from different states was observed. Participants also highlighted within state variation as a challenge for service delivery.

“*The biggest issue is regional variation*. *Depending where you live determines the service level you receive”*. *(Child and Adolescent Psychiatrist 1)*

In addition, the reduction in funding for public mental health services and the transfer of responsibility for service delivery to non-government organizations (NGOs) has impacted service delivery.

“*I think we have to accept that the two systems are going to run side-by-side*, *but if we want to have a good public system we have to fund it properly*.*” (Pediatrician 7)*

“*We've had the state run services divesting their care provision to NGOs who are highly variable in their practise*. *And then of course the change to the state*, *federal state disability agreement and the abolition of state provided disability services and transferred to the NDIS*. *So consequently it's become much more fragmented at the moment*, *the reason is*, *it's going through all these major changes”*. *(Child and Adolescent Psychiatrist 8)*

“*Our community has probably never been in more need and we just have a lack of services*, *that are free*, *that are available for the community”*. *(Psychologist 5)*

Clinicians identified that while teleconferencing could be a useful tool for supporting families (particularly in rural and remote areas) psychologists are unable to assign a MBS item number to teleconferencing consultations, in contrast to pediatricians and psychiatrists.

“*People have to travel three hours for a one hour appointment and then three hours home with an autistic kid in the back seat*. *It's a disaster*. *I can't do a Skype consult with parents using a mental healthcare plan…… they have to be in the room*”. (*Psychologist 3)*

Participants identified several situations where silos (lack of coordination, shared resources, etc.) exist as a barrier to optimal care. Funding is siloed, conditions are siloed and research activities are siloed.

“t*hat’s been also a big barrier that*, *that whole discipline of [ADHD] research and clinical practice has almost developed in complete parallel to Autism and yet we know that 3/4 of kids will have ADHD and a 1/3 of children with ADHD will have Autism*, *that’s crazy*.*” (Psychologist 1)*

Clinicians identified that whether a condition falls within the disability sector or healthcare sector impacts care coordination and management as the two sectors do not usually integrate.

“*……governments work in silos and it’s very much about the type of services that are definitely not education and are definitely not health*. *So*, *again*, *it would be ideal if somehow or other the government departments responsible…*..*could look at ways of breaking down these interdepartmental silos*. *I know in [xxxxx] there have been some attempts to do that but it remains difficult*.*” (Child and Adolescent Psychiatrist 8)*

Even within the healthcare sector conditions were identified as either falling within mental health or developmental paediatrics.

“*so I actually don’t think mental health services should be siloed and exclude children who have co-morbidities and disabilities*, *or ASD so this is the thing as well*, *if they have got an intellectual disability or ASD*, *then psychiatry won’t see them”*. *(Pediatrician 3)*

Participants highlighted that systems of care also depend on the clinicians involved. Availability and quality of providers can be variable, particularly in rural locations and frequently.

“*It’s a hit and miss thing depending on who they are seeing” (Psychologist 1)*

“I think from my experience working in more remote parts of [xxxxx] is that it was really dependent on who the actual teams were who were on the ground.” (Child and Adolescent Psychiatrist 6)

#### Financing structure factors

The introduction of the NDIS is a national policy impacting children with complex needs. While the NDIS was identified as a substantial change, the impact of the NDIS for specific conditions and programmes is uncertain.

“*I would prefer the children I look after with ADHD not to see themselves as having a disability*, *but I know that there are families who have access to NDIS with that diagnosis*. *I'm waiting to see whether that becomes standard or not”*. *(Pediatrician 7)*

The relationship (or lack thereof) between the healthcare sector and the disability sector was discussed.

“*I think one of the real challenges within service provision within this country at the moment is going to be how we integrate and inter-relate with NDIS services”*. *(Pediatrician 13)*

Participants expressed concerns about the implications of the NDIS model. Parents plan support packages for their children with the assistance of NDIS planners who may or may not have experience with the conditions being discussed. Concern was expressed that healthcare professionals are not required to be involved in designing treatment and care for children.

“*I think in the next 10 years there's a chance for it to evolve into something positive rather than not*, *so I think that's an opportunity*, *but I think at the moment it seems*, *at least to the pediatricians on the ground…*..*as though we haven't been involved at all in that NDIS discussion and we're not being invited to the table when care is being planned much at all”*. *(Pediatrician 7)*

“*the parents don’t even have to give our report to the provider*, *they don’t give the medical report to the NDIS planner*.*” (Pediatrician 8)*

Some participants questioned whether mental health conditions would have sufficient support under the NDIS.

“*…*..*by going down the path of an NDIS*, *which has other strengths that goes*

with it, but it really diminishes the value of understanding the particular, the particularities around autism”. (Health system manager 1)

However, other participants felt that the healthcare sector would become more involved with time and that it would improve outcomes for children.

“*…*..*a huge opportunity in Australia to improve the way we provide services particularly to improve things to a more functional level for these kids” (Psychologist 1)*

In addition, existing Commonwealth government funded support as part of the Helping Children with Autism funding (HCWA) will be phased out. Although support will be transitioned to the NDIS, uncertainty exists about who will retain support and how this will be managed.

“*the DSM 5 criteria changed so there’s been some issues around that*, *and so both that and the NDIS*, *given the criteria are different*, *both of them…*.. *from what I’ve heard have led to lower levels of eligibility so we know that a lot of kids who are eligible under Helping Children With Autism*, *for example*, *are not eligible under the NDIS for services*.*” (Psychologist 9)*

#### Parent and family factors

Participants highlighted the key role that parents play in the ongoing management and coordination of care for children with complex mental health needs.

“*basically up to parents and to some extent the profile of the child as to who people will access*.*” (Pediatrician 2)*

However, challenges for parents were identified.

Clinicians felt that many areas of children’s care are substantially influenced by the capacity of parents to advocate on behalf of their child.

“*families who are good self-advocates tend to have the best outcomes because so much is reliant on good advocacy skills by a parent or guardian*.*” (Pediatrician 1)*

Concerns were expressed about how parents with limited advocacy skills manage the complex process of accessing support and funding.

“*the truth is there will never be a one-system-fits-all*. *So I despair almost at the difficulties of people at low socioeconomic status and English as a second language in being able to compete with the articulate*, *tertiary-educated parents who can convince the planners to give them mega-packages*.*” (Pediatrician 4)*

Consistently, participants identified that the system of care is hard to navigate for parents (and clinicians). A lack of coordinated services means parents require skills to understand the system and access services.

“*parents find it extremely confusing and constantly I find families that I feel like*, *you shouldn't have to reinvent the wheel*.*” (Psychologist 4)*

“*They [often] see the same practitioner by default really*. *Someone who’s in their area*, *who bulk bills*, *who their child will talk to*.*”(Child and Adolescent Psychiatrist 5)*

The issue identified by participants as most problematic was transition to adult care. Many clinicians identified that once children leave the pediatric system, continuity of care was very limited and adversely impacted by out-of-pocket costs to the family.

“*…*..*at the moment that’s the biggest point of weakness that I see in our system*.*” (Pediatrician 2)*

“But it certainly leaves young adults high and dry, and often, families are required to transition to private adult services with a marked decline in participation because there’s a cost involved. So it’s a terrible weakness, a terrible gap”. (Pediatrician 10)

Participants identified challenges and frustrations where Government funding was provided for services and therapies which did not have an evidence base. This placed the responsibility on either the treating clinician or the parents to identify and implement evidence-based treatments which can be difficult in the internet era.

“*if government money was more tightly linked to evidence*, *families would spend a lot less time and effort and money on interventions that are likely to do nothing at all for them*.*” (Pediatrician 7)*

“*…*..*and really now children or their families can receive a whole range of different interventions for autism through the helping children with autism funding which are not based on an agreed policy on early intervention*,*…*..*It's very much open to the whim and wish of parents and perhaps their advisors*.*” (Child and Adolescent Psychiatrist 8)*

### Aim 2. Enablers to access and optimal care

Participants identified several factors which could improve access to care.

#### Healthcare system factors

While participants identified challenges providing services in regional locations they also suggested that benefits existed which did not occur in urban locations. These include the physical proximity of local professionals and the relationships that can develop as a result.

“*for a pediatrician who works in a smaller community it’s possible to know all the networks*, *be familiar to set up a reasonably good system for children”*. *(Pediatrician 2)*

“*…*..*on the other hand some rural communities have better integration of support services*. *All the workers from different agencies all know each other because everybody knows each other in the town*. *Whereas in the middle of [major city] I wouldn't know all the people working in autism within a kilometre from where I am*. *So I think it's got its pros and cons*.*” (Child and Adolescent Psychiatrist 9)*

Clinicians identified models of care in physical health conditions which were effective in reducing the burden for families and suggested the same strategies may be valuable for supporting families of children with complex mental health conditions.

“*They have nurse navigators for diabetes*, *but we're getting them for autism*, *which is a big leap forward*.*” (Pediatrician 3)*

“*the autism advisors have been a decided success nationally*, *whereby there has been a regular place where people can get information and a pathway for how they can work their way through*, *at any point*, *keep coming back to these people…*.. *It has made*, *particularly*, *a big impact on school life and early intervention life*. *So that*, *that’s probably the most*, *one of the more successful pathways that has been established in the field of disability*.*” (Health system manager 1)*

While participants consistently highlighted variation between states, they also identified that there are models of care operating within some states were perceived to be effective in reducing fragmentation in service delivery.

“*in terms of a model*, *would be something like a Child-Development Unit where everybody was physically sited together and working together*.*” (Pediatrician 7)*

It was also recognised that this is a complex and international issue rather than a specific fault within the Australian healthcare system.

“*the only thing I think I learned at [overseas institution] is that we were doing a reasonable job*.*” (Pediatrician 13)*

#### Financing structure factors

Despite recognising some system level limitations, universal health insurance through the MBS is considered to underpin access to care and is seen as a key facilitator of access to both primary and secondary care.

“*I do believe the Medicare system here is actually overall not broken*, *it’s a good system*, *it’s been shown to work for many*, *many different medical conditions and I think everyone knows how it works……I think the Medicare system is a good structure in keeping the primary care provider involved in the loop*.*” (Pediatrician 5)*

#### Parent and family factors

Informed and capable parents were recognised as major enablers of optimal care for children with complex mental health conditions. This is particularly given the complexity of the healthcare system for these children as it currently operates.

“*They can be a major advocate for a child to pull all the different services together*.*” (Pediatrician 1)*

“*the coordination of care seems to rest with parents and families so the more capable parents seem to set up really nice systems of care for their children*.*” (Pediatrician 2)*

### Aim 3. Components of an optimal model

In an environment where healthcare funding is limited and children require support in multiple settings, empowering parents to manage their children within the healthcare, disability and education systems was identified as key to improving outcomes for children with complex needs.

“*It would be really good if there was a standard of what you needed to provide and how much care the child needed* … *But of course every child is so independent and it is so dependent upon their families capacity to provide services themselves*.*” (Psychologist 4)*

Clinicians recognised the key role of parents and the fact they had more involvement and knowledge of their children’s needs. The family has much more contact with children therefore increasing capacity for impacting care for their child.

“*What I can do in half an hour a month is nothing compared to what a parent can do*.*”(Pediatrician 9)*

“*Parents are the main therapists*. *That’s what we try to do*, *we try to empower parents that drive this and of course some parents can’t but… because the parent's there all the time*, *the parent knows the child better than an Autism practitioner”*. *(Psychologist 3)*

Empowering parents to support their children was seen as critical to ensuring continuity of care. This means providing tools, resources and support.

“*…*..*it is something they can then use as a talking point with someone who is going to provide them with ongoing care*, *so a pediatrician or an intervention worker or whoever person is facilitating all that advocacy in services and they can kind of just use that*.*” (Pediatrician 2)*

Clinicians identified that purposefully working with other disciplines is key to improving outcomes in Australia. Greater cooperation between providers would simplify service delivery and streamline service delivery.

“*I think again that the multidisciplinary approach is really helpful*. *To be able to utilise different skill sets and do assessments across a range of domains is really important*.*” (Child and Adolescent Psychiatrist 6)*

“*In my multidisciplinary team …*..*we discuss with parents what they feel they need then perhaps amongst ourselves; we discuss where the child is at and what we think might be ideal and then we go back and try and integrate those two sets of ideas and then create a plan from there*.*” (Pediatrician 2)*

In addition to multi-disciplinary teams, participants identified that due to the high level of comorbid conditions greater cooperation between treating clinicians is necessary and beneficial. Where this is occurring, clinicians felt that outcomes were improved.

“*we meet four weekly with the psychiatrist and their social workers and psychologists*..*…that one on one chat sort of helps to break down those barriers*.*” (Pediatrician 13)*

“*I think the barriers are the lack of everybody being sited together and the fact that all of these different professions and craft groups work in their own little bubble*.*” (Pediatrician 6)*

Strengthening education systems was also a key theme. Participants recognised that children and adolescents spend a considerable amount of time in the education system.

“*……*..*they did set up what's called Ed-Link positions*. *Child and youth positions that worked with schools*. *Young people spend most of their time there*.*” (Child and Adolescent Psychiatrist 6)*

“*I think that the more that people who are engaged and that are involved with these kids on a day to day basis*, *such as the school and the parents*, *the more that we can actually have interaction between those groups*, *and everyone knows where they're coming from*, *the better it is*.*” (Pediatrician 12)*

Co-location and integration of services was identified as beneficial to children, families and clinicians. Benefits over and above physical proximity included coordination and integration of care for these children.

“*Get them in the same room is probably a start*. *Sharing a tea room would be a start*. *I gratefully remember sharing the tea room*.*” (Pediatrician 3)*

“*… one where people were seen by the person who was the most expert in their field in regards to that problem*, *and where there was medical input as well as non-medical input because I think…*..*that's really important to have everybody there…*..*” (Pediatrician 7)*

“*a Child-Development Unit where everybody was physically sited together and working together so that there had to be some consensus reached about things like*, *what is good intervention and what is not*, *and what's a complete waste of people's time and money and what's not*. *At the moment*, *that's not integrated at all*, *and families just go off on their own and figure out whose story they want to believe in terms of what they should do next”*. *(Pediatrician 7)*

Given the distances in Australia, being physically situated in close proximity can be difficult. However, other mechanisms were discussed including the role of technology in case management. These options can be particularly valuable in bringing together all support services, including education.

“*we're videoconferencing schools actually to have them understand what's required and give them support in helping those kids at school*. *It's not difficult at all*, *you just have to be used to it*.*” (Pediatrician 12)*

Increasing the level of knowledge within the healthcare workforce for the management of children with ADHD and Autism will strengthen the overall system of care. Capacity building by incorporating knowledge into training for both pediatric and adult specialists (as well as GPs) was seen as an opportunity to build a more sustainable knowledge base.

“*I think that there should be an expectation that all clinicians who go and work in child and adolescent mental health services receive in-service training about working with intellectually /developmentally disabled people*, *as they receive training about working with different cultures and different linguistic groups*.*” (Child and Adolescent Psychiatrist 5)*

In addition, succession planning by the professions is required, particularly in smaller regional and rural areas.

“*I think stabilising and maintaining the ageing workforce [and]……bring in new and younger practitioners in partnership with those people if at all possible*, *and then to actually build long term structures…*..*That's what's necessary*.*” (Pediatrician 9)*

## Discussion

This is the first Australian study to describe a national view of systems of care for children with complex mental health conditions from the perspective of key clinicians involved in their management. Our study identified barriers and enablers to optimal care as well as components of models of care likely to improve outcomes for children and families.

### Barriers and enablers of access to mental health care

Key barriers included systemic and structural issues including the Federal/state funding divide, the pluralistic structure of healthcare in Australia and the challenges of delivering healthcare in diverse geographic settings. Limitations in use of specific MBS item numbers by different clinicians, e.g. lack of telehealth MBS item numbers for psychologists and case management MBS item numbers for other professions, were also considered barriers. Clinicians also highlighted reduced funding for public mental health services and the challenges of navigating the system for families and clinicians including the lack of integration between the health and disability sectors. Consistent with international research[34] transitioning from child and adolescent services to adult services was identified as the most compromised area of care.

Unlike previous research, this study found that regional models were an enabler of access and metropolitan models should consider the enabling factors identified. Despite limitations, overall the MBS was perceived as an enabler. The ability of the family to act as advocates and care co-ordinators was highlighted as a vital enabler of accessing care, consistent with previous evidence suggesting parents can be powerful agents for change.[35] The benefit of learning from models that are working successfully either across the same conditions in other states or in other conditions (e.g. diabetes) was also identified.

### Potential solutions

Similar to previous research, clinicians identified that empowering parents to advocate for and deliver care is key.[36, 37] Children with complex mental health needs typically require support in a range of settings (e.g. disability, health, education) where parents are generally the common thread and the most consistent influence in the child’s life. Parent mediated interventions have been shown to be effective in improving language skills and reducing autism symptoms in children. [38,39] Peer-to-peer support by parents has been used successfully to support the child mental health workforce. [40] Peer support networks (either face to face or online) help parents navigate the system and advocate for their child.[41, 42] In addition, expansion of existing support structures to assist parents to navigate the health care system e.g. Autism Advisor roles, and education about evidence-based tools and resources available in the community could support parents to improve care quality and coordination.

Systems of care that focus on streamlined, coordinated services and cooperation between parents, health, disability and education sectors was identified as another key component in improving outcomes. Multidisciplinary solutions where medical and non-medical services work together is a critical component of an ideal model of care. Underpinning coordination and multidisciplinary solutions is the need for strengthening workforce knowledge through improved workforce planning, succession planning and specific training for treating complex mental health conditions.

Co-location of services supports the provision of optimal care. The relationships and networks that can develop through co-location of services was perceived to enhance the opportunity for multidisciplinary and coordinated services. However, to achieve improved outcomes in the long term, silos identified by clinicians between professional groups, government departments and sectors need to be broken down. The benefits of co-location can occur by default in rural environments, highlighting the value of looking at strengths as well as challenges of different settings.

### Strengths and limitations

Our study had several strengths. This was the first Australian study to seek and discuss clinician’s views in key professions that manage children with complex mental health conditions. The project involved Australia wide multi-disciplinary representation from metropolitan and rural areas. The interview guide was based on an international best practice survey for optimum care for children with complex needs but adapted for the Australian context.[43] There were some limitations to our study. General practitioners were not surveyed due to limited funding. The sample was predominantly experienced clinicians who may have seen several attempts at a policy level to improve outcomes with limited success. However, the sample did include some more junior specialists and their views did not differ from those of more experienced practitioners. The national scope of the study put inevitable restrictions on the extent to which the issues under consideration could be explored in more depth at state/territory, regional or local levels.

## Conclusion

Many challenges exist for the delivery of optimal care for children with common, complex mental health conditions across Australia. Restructuring service delivery into more streamlined, multidisciplinary care requires system level changes by government departments and professions involved. Given the complexity of implementing these types of system level changes, empowering parents to advocate for services and understand how the systems work, is likely to have the most impact in the short term. Future research needs to explore how best to do this (e.g. online, via care coordinators etc.), including evaluating effectiveness and cost-effectiveness of such approaches. Clinicians also identified that care needs to be multi-disciplinary, integrated, preferably co-located and should involve the education sector if Australian children with complex mental health conditions are to realise their potential. Developing, implementing and then evaluating the impact of integrated care models on child mental health, care access and costs is a future research priority.

## Supporting information

S1 File Scenario and questionaire ADHD final. (DOCX)

S2 File Scenario and questionaire Autism final. (DOCX)

S3 File Scenario and questionaire ADHD + Autism final. (DOCX)
