# Supplementary material for: Strengthening care for children with complex mental health conditions: Views of Australian clinicians
Source: PLoS One. 2019 Apr 2;14(4):e0214821. doi: 10.1371/journal.pone.0214821 (PMC6445417; doi:10.1371/journal.pone.0214821)
Supplement: S2 File — (DOCX) [file pone.0214821.s002.docx]

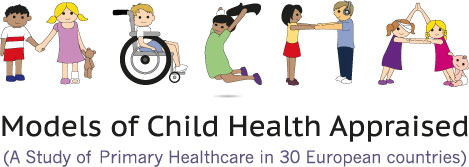


**WP2: Safe and Efficient Interfaces of Models of Primary Health Care with Secondary, Social and Complex Care**

**Task 2: Complex Care Survey (enduring mental health issues)**

Dear Colleague,

The research team on Task 2 of WP2 MOCHA are to provide a reliable and comprehensive analysis of the current approach to managing the care of children with complex care needs at the acute/community/primary interface. This is to generate an evidence base of clear and accessible information concerning current provision of care. We are seeking your feedback on a number of specific scenarios where a child has an enduring and complex health condition. In this case, we are looking at Autism.

To answer these questions, the task team suggest that you should seek to find **someone who understands the complexity outlined in the scenario.**  This person could be a professional placed in the mental health service or social service, advocacy group, diagnosis specific actions group, and / or primary care personnel. In addition national leads from a Department of Health and /or a Department of Education may be relevant.

To answer the questions about family engagement, you should ideally seek the views of a patient advocacy group. Please answer as many questions as you can, giving examples or elaborating if you would like.

Included with these questionnaires is a glossary of terms.

The survey consists of two sections: Section 1. Systems of Care for Children with Complex Care Needs and Section 2. European Survey of Change.

At the end of the survey we ask that you to provide us with the names and contact details of all those who provided feedback, so that we may acknowledge their contribution in our final report.

*Thank you*

**Case scenario – Autism**

Autism or Autism Spectrum Disorder (ASD) is a set of pervasive developmental disorders, emphasised by the early onset of a triad of features: impairments in social interaction; impairments in communication; and restricted, repetitive, and stereotyped behaviour, interests, and activities (Lai, Lombardo & Baron-Cohen, 2013; World Health Organisation, 2016).

Mark is 2 years and 5 months old and has been newly diagnosed with Autism. His development was considered normal up to the age of 15 months. He communicated with babble, held eye contact and reached motor skill milestones as expected. At 18 months Mark’s development began to stagnate, he experienced language regression and had difficulty maintaining eye contact. His parents described him as socially withdrawn, lacking interest in interacting with peers and having mannerisms with his hands. He also has a tendency to run askew, with his hands turned backwards. Currently, at diagnosis, Mark has been assessed as having severe and pervasive difficulties in all areas of the autistic spectrum. He is not developing age appropriate expressive or receptive language. In addition, his ability for social interaction as well as functional and symbolic play is found to be deviant and not age appropriate. This is considered stereotypical of Autism. Mark’s gross and fine motor skills are also affected.

His future health and social care should be delivered in an environment where specialists with expertise in respect to Autism can provide a care plan to support his development. It is also advised that his parents receive guidance in meeting Mark’s needs in the family setting. Mark’s condition is irreversible and he is expected to receive lifelong care and treatment, aimed at preventing further regression and develop new skills. The care will be provided by a multi-disciplinary team consisting of psychiatrists, psychologists, physiotherapists, occupational therapists, special community child and youth workers, social care workers, home care nurses and respite care services.

There is a high degree of heterogeneity across the Autistic Spectrum. Some children with Autism will experience relatively little impairment and may not require the same level of expert care as Mark. It is expected, however, that the majority of children with Autism, will require substantive care for their enduring complex care needs.

**SECTION 1: SYSTEMS OF CARE FOR CHILDREN WITH COMPLEX HEALTH NEEDS**

This section of the survey includes questions on the care of a child with AUTISM in your state, adapted from the *Standards for Systems of Care for Children and Youth with Special Health Care Needs* (AMCHP and Lucile Packard Foundation, 2014). Please complete each section guided by the scenario provided of Mark and his family.

To ensure alignment in translation across the EU countries, some of the questionnaire keywords are specified here:

**Process:** A set of interrelated activities, which transform inputs into outputs.

**Procedure/guideline**: Description of actions done in a certain way.

**Policy:** A plan or course of action of decision makers at macro and meso-level to influence and determine decisions, actions, and other matters. This refers to a legal framework.

**A system:** A set of methods, procedures, arrangements, and activities that are carried out to form a unity or to meet a common goal.

This survey will, among other issues, deal with the integration of health and social care services. The organisation and integration of these services will vary substantially between countries. If there is a framework in your state for the integration of health and social services, please comment in the open-ended questions, as to whether the health and social needs of children with Autism are adequately integrated or fragmented.

| SCREENING, ASSESSMENT, AND REFERRAL | | | | | |
| --- | --- | --- | --- | --- | --- |
| In my state, there are policies and/or procedure(s) in place to assure that a child like Mark receives *ongoing* preventative care screening and developmental checks with regard to their Autism diagnosis. | | Policies  Yes  No | *If yes,* please provide reference(s) for the policy or policies. | If yes, who starts this process? | |
|  |  | Procedure(s)  Yes  No | *If yes,* please provide reference(s) for the procedure(s) | If yes, who starts this process? | |
| *If there are policies and/or procedure(s) in place to ensure that a child like Mark receives ongoing preventative care screening and developmental checks with regard to their Autism diagnosis*   1. please list the type of examinations provided | |  | | | |
| 1. please name the healthcare providers that conduct these examinations (e.g. examination that screens for mental health comorbidity) | |  | | | |
| 1. please name the social care providers that conduct these examinations (e.g. social well-being screening) | |  | | | |
| In my state, there are policies and/or procedure(s) in place to document and communicate the results of such screening to   1. all care services (e.g. general practitioners/ primary care physicians/ paediatrician, community nurses, school, secondary care) caring for the child? | | Policies  Yes  No | *If yes,* please provide reference(s) for the policy or policies. | *If yes,* who is in charge of the  communication process? | |
|  |  | Procedure(s)  Yes  No | *If yes,* please provide reference(s) for the procedure(s) | *If yes,* who is in charge of the  communication process? | |
| 1. the child’s parent(s) / guardian(s)? | | Policies  Yes  No | *If yes,* please provide reference(s) for the policy or policies. | *If yes,* who is in charge of the  communication process? | |
|  |  | Procedure(s)  Yes  No | *If yes,* please provide reference(s) for the procedure(s) | *If yes,* who is in charge of the  communication process? | |
| In my state, there are policies and/or procedure(s) in place to assess family capacities (e.g. knowledge and ability) that may influence providing care for the child with Autism. | | Yes  No | If yes, please provide reference(s) for the policy or policies | If yes, please list who is in charge of  this assessment | |
| Have you any further comments in relation to the screening, assessment, and /or referral of children with Autism in your state? | |  | | | |
| ACCESS TO CARE | | | | | |
| There is a system in place in my state to identify   1. all of the healthcare providers who care for children with Autism. | Yes  No | | *If yes,* who is in charge of this? | | |
| 1. all of the social care providers who care for children with Autism. | Yes  No | | *If yes,* who is in charge of this? | | |
| In my state there is an agreed multi-disciplinary guideline of care for children with Autism | Yes  No | | *If yes,* who is in charge of this? | | |
| In my state formal training on how to best care for children with Autism is provided to the parent(s) / guardian(s). | Yes  No | | *If yes,* which type of care are the parent(s) / guardian(s)  to provide? | | |
| *If parent(s) / guardian(s) receive formal training with regard to the care of children with Autism*  are there policies and/or procedures in place to ensure that they have the capacity (e.g. knowledge and ability) to incorporate this into their child’s daily routine | Policies  Yes  No | | *If yes,* please provide reference(s) for the policy or policies. | | |
|  | Procedure(s)  Yes  No | | *If yes*, please provide reference(s) for the procedure(s) | | |
| Please provide a list of the statutory primary and secondary healthcare providers caring for children with Autism in your state (For example Child and Adolescent Psychiatry or paediatric units). |  | | | | |
| Please provide a list of the voluntary healthcare providers caring for children with Autism in your state (For example respite services etc.) ^[[1]](#footnote-1)^. |  | | | | |
| Please list the professions responsible, in your state, for providing general healthcare services to children with Autism in the community after treatment for an acute behavioural episode (For example general practitioner). |  | | | | |
| Please list the professions responsible, in your state, for providing social care services to children with Autism in the community after treatment for an acute behavioural episode (For example specialised social care workers). |  | | | | |
| Are there policies and/or procedure(s) in your state, which provide children like Mark and his family an overview of the interface between the health care and social care system responsible for Mark’s primary and secondary care? | Yes  No | | | *If yes,* please provide reference(s) for  the procedure(s) | |
| *In my state …* |  | | |  | |
| … there are procedures in place to ensure that children like Mark are treated by the same healthcare provider / team of healthcare providers every time. | Yes  No | | | *If yes,* please provide reference(s) for  the procedure(s) | |
| … there are procedures in place to ensure that children with Autism are treated by the same social care provider / team of social care providers every time. | Yes  No | | | *If yes,* please provide reference(s) for  the procedure(s) | |
| In my state |  | | |  | |
| … a child with Autism can access primary care regardless of care provided in secondary care and vice versa. | Yes  No | | | *If yes,* please provide reference(s)  that supports your answer | |
| … there are policies and / or procedures in place, which *facilitate* or *hinder* children with Autism in accessing primary and secondary care simultaneously. | Yes  No | | | Facilitators | Barriers |
| … there is transportation to care service for children with Autism (e.g. access to special school, health assessment, etc.) in my state is provided by | 1. the child’s parent(s) / guardian(s) with support from the state | | | Yes  No | |
|  | 1. the child’s parent(s) / guardian(s) without support from the state | | | Yes  No | |
|  | 1. the healthcare provider | | | Yes  No | |
|  | 1. the social care provider | | | Yes  No | |
| … there are policies and / procedure(s) in place to ensure that all information provided to families of children with Autism is linguistically appropriate. | Policies  Yes  No | | *If yes,* please provide reference(s) for the policy or policies. | | |
|  | Procedure(s)  Yes  No | | *If yes*, please provide reference(s) for the procedure(s) | | |
| In my state, there are policies and / procedure(s) in place to assure that all information provided to families of children with Autism is culturally appropriate. | Policies  Yes  No | | *If yes,* please provide reference(s) for the policy or policies | | |
|  | Procedure(s)  Yes  No | | *If yes*, please provide reference(s) for the procedure(s) | | |
| Have you any further comments in relation to the accessibility of care for children with Autism in your state? |  | | | | |
| CARE COORDINATION | | | | | |
| *In my state,* | |  |  | | |
| …there are policies and/ or procedures promoting access to care coordination for the child diagnosed with Autism. | | Policies  Yes  No | *If yes,* please provide reference(s) for the policy or policies | | |
|  |  | Procedure(s)  Yes  No | *If yes,* please provide reference(s) for the procedure  or procedures | | |
| … there is a specific care pathway for children with Autism | | Yes  Partly  No | *If yes,* please provide reference(s) to support your answer | | |
| *If there is a care pathway for children with Autism are there policies or procedures that clearly describe each provider’s role in a care pathway for children with Autism* | | Yes  No | | | |
| … there are procedures in place for ensuring a written personalised plan of care for children diagnosed with Autism. | | Yes  No | *If yes,* please list who is responsible for this? | | |
| … there is specific integration procedure and/ or policy of pathways for children with Autism, which combines primary and secondary care | | Yes  No | *If yes,* please provide reference(s) for the procedure(s) | | |
| *If there is specific integration procedure and/ or policy of pathways for children with Autism, which combine primary and secondary care, does this facilitate continuity?* | | Yes  No | *If yes,* how? If possible please link to the evidence that  support your answer | | |
| …the written personalised plan of care for a child with Autism is developed in consultation with   1. the child’s parent(s) / guardian(s) | | Yes  No | *If yes,* who starts it? | | |
|  |  |  | *If yes,* who is in charge? | | |
| 1. Other healthcare professionals | | Yes  No | *If yes,* who starts it? | | |
|  |  |  | *If yes,* who is in charge? | | |
| 1. Social care professionals | | Yes  No | *If yes,* who starts it? | | |
|  |  |  | *If yes,* who is in charge? | | |
| …the personalised written care plan for a child like Mark integrates the following ;   1. developmental assessments | | Yes  No | | | |
| 1. mental health comorbidity assessments | | Yes  No | | | |
| 1. physical health | | Yes  No | | | |
| 1. oral health | | Yes  No | | | |
| 1. vision health | | Yes  No | | | |
| 1. hearing assessments | | Yes  No | | | |
| 1. social health | | Yes  No | | | |
| … hospital(s) that provide mental health care for children such as Mark (both outpatient and inpatient) have a discharge planning coordinator responsible for organising the transition of a child with Autism from the hospital setting to the child’s home or another community based setting. | | Yes  No | | | |
| Autism often is associated with a high degree of comorbidity. Can you provide information on how children with Autism are cared for in your state in case of comorbidity? | |  | | | |
| Have you any further comments in relation to the coordination of care for children with Autism in in general your state? | |  | | | |
| COMMUNITY-BASED SERVICES AND SUPPORT | | | | | |
| *In my state,* | |  |  | | |
| … community-based services (e.g. local initiatives or voluntary organisations) are a key component of the care provided to children like Mark. | | Yes  No | If yes, who coordinate the community-based care with the  hospital-based care | | |
| …family advocacy groups are involved in making recommendations to home and community-based services for children with Autism. | | Yes  No | | | |
| … the parent(s)/guardian(s) and siblings of children with Autism have access to professional psychosocial support. | | Yes  No | *If yes,* who starts this process? | | |
| …respite care is available for the parent(s) / guardian(s) children with Autism | | Yes  No |  | | |
| *If respite care is available, is it provided by* | | **State**  Yes  No  **Voluntary sector**  Yes  No  **Private sector**  Yes  No | *Please list the main provider for respite care in your state* | | |
| … children with Autism are offered individually tailored educational and / or training interventions or accommodations in response to their specific requirements. | | Yes  No |  | | |
| Have you any further comments in about the community based services and supports for children diagnosed with Autism in your state? | |  | | | |
| FAMILY PROFESSIONAL PARTNERSHIP | | | | | |
| *In my state parent(s) / guardian(s) of children with Autism are* | |  |  | | |
| … invited to participate in the development of policies and procedures affecting their children. | | Yes  No |  | | |
| … included in national quality improvement initiatives for Autism. | | Yes  No |  | | |
| … involved in the review of patient and family information material on Autism for the public, to ensure cultural and/or linguistic competency. | | Yes  No |  | | |
| Have you any further comments in about family professional partnerships relating to the care of children with Autism in your state? | |  |  | | |
| TRANSITION INTO ADULTHOOD | | | | | |
| In my state, there are policies and procedure(s) in place to ensure continuity of care for adolescents with Autism transitioning to adult specialists and community supports. | | Yes  No | *If yes,* please provide reference(s) for the policies or procedures. | | |
| Please list the any barriers to continuity of care for adolescents with Autism transitioning to adult care services in your state. | |  | | | |
| Have you any further comments in relation to the transition of adolescent with Autism to adult care services? | |  | | | |
| QUALITY ASSURANCE | | | | | |
| *In my state,* | |  |  | | |
| …there are quality assurance policies and/or procedures for service providers caring for children with Autism. | | Policies  Yes  No | *If yes,* please provide reference(s) for the policy or policies. | | |
|  |  | Procedure(s)  Yes  No | *If yes,* please provide reference(s) for the procedure or procedures | | |
| …data is collected on the experience of care for children with Autism from the perspective of   1. the parent(s) / guardian(s) | | Yes  No | *If yes*, who undertakes the collection and analysis of these data? | | |
| 1. the siblings | | Yes  No | *If yes,* who undertake the collection and analysis of these data? | | |
| 1. primary health and social care professionals | | Yes  No | *If yes,* who undertake the collection and analysis of these data? | | |
| Have you any further comments about ensuring that children with Autism in your state receive quality care? | |  |  | | |

**SECTION 2: EUROPEAN SURVEY OF CHANGE**

This section of the survey aims to provide a ‘barometer’ of the current situation of integration of care for children with complex care needs. You are asked to answer a number of questions to help us gain an understanding of current issues and debates on the issues of complex care for children with AUTISM in your state.

Please answer ***all*** the questions as accurately as possible and make use of the space for comments to clarify your information and to describe distinct characteristics of services/health organisations in your state.

1. **QUESTIONS ON THE CURRENT STATE OF DEVELOPMENT OF SERVICES FOR CHILDREN DIAGNOSED WITH AUTISM**
   1. Please list the three most significant changes that have taken place in the integration of care services for children diagnosed with Autism in your state in the last five years?

(i)

(ii)

(iii)

- 1. How would you evaluate the overall progress in the integration of care services for children with Autism in your state in the last five years? Please indicate with an ‘x’ the one statement that best describes this progress from the three options given below. Since January 2010 the integration of care for children with AUTISM.
     1. has improved
     2. has remained very much the same
     3. has got worse
  2. Please list in order of importance the three main ***barriers*** to the integration of care services for children with Autism in your state at the present time:

(i)

(ii)

(iii)

- 1. Please list in order of importance the three main ***opportunities*** to the integration of care services for children with Autism in your state at the present time:

(i)

(ii)

(iii)

1. **QUESTIONS ON POLICY AND SOCIO-CULTURAL ISSUES**
   1. What strategies have been used to improve political awareness and government recognition of the need for integration of care services for children with Autism in your state in the last five years?
   2. Have there been any special funding initiatives for this group by government, private/voluntary care organisations, Non-Governmental Organisations, Organizzazione Non lucrativa di Utilità Sociale (ONUS),or donors in the last five years?
   3. ~~Has there been any involvement between your state and the European Union in relation to initiatives on managing the integration of care of children with AUTISM in the last five years?~~
   4. Has there been any major public discussion, debate or controversy about the needs of children with Autism in your state in the last five years?

Additional Questions

Thinking about mental health more broadly,

1. In an ideal world how would you like to see mental health services offered?
2. How do you think that fits with what is happening now?
3. What could we do now to take it closer to the ideal?
4. Do you think there is a difference between rural and urban areas in terms of the questions that have been asked, e.g. at a policy and procedural level

1. This list may not be exhaustive [↑](#footnote-ref-1)
