# Supplementary material for: Strengthening care for children with complex mental health conditions: Views of Australian clinicians
Source: PLoS One. 2019 Apr 2;14(4):e0214821. doi: 10.1371/journal.pone.0214821 (PMC6445417; doi:10.1371/journal.pone.0214821)
Supplement: S3 File — (DOCX) [file pone.0214821.s003.docx]

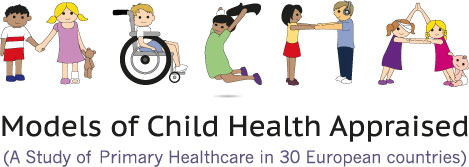


**WP2: Safe and Efficient Interfaces of Models of Primary Health Care with Secondary, Social and Complex Care**

**Task 2: Complex Care Survey (enduring mental health issues)**

Dear Colleague,

The research team on Task 2 of WP2 MOCHA are to provide a reliable and comprehensive analysis of the current approach to managing the care of children with complex care needs at the acute/community/primary interface. This is to generate an evidence base of clear and accessible information concerning current provision of care. We are seeking your feedback on a number of specific scenarios where a child has an enduring and complex health condition. In this case, we are looking at **Attention Deficit Hyperactivity Disorder (ADHD) and Autism**.

To answer these questions, the task team suggest that you should seek to find **someone who understands the complexity outlined in the scenarios.**  This person could be a professional placed in the mental health service or social service, advocacy group, diagnosis specific actions group, and / or primary care personnel. In addition national leads from a Department of Health and /or a Department of Education may be relevant.

To answer the questions about family engagement, you should ideally seek the views of a patient advocacy group. Please answer as many questions as you can, giving examples or elaborating if you would like.

Included with these questionnaires is a glossary of terms.

The survey consists of two sections: Section 1. Systems of Care for Children with Complex Care Needs and Section 2. European Survey of Change.

At the end of the survey we ask that you to provide us with the names and contact details of all those who provided feedback, so that we may acknowledge their contribution in our final report.

*Thank you*

**Case scenario – Attention Deficit Hyperactivity Disorder**

Attention Deficit Hyperactivity Disorder (ADHD) is a childhood-onset neurodevelopmental disorder characterised by developmentally inappropriate and impairing inattention, motor hyperactivity, and impulsivity, with difficulties often continuing into adulthood (Thapar & Cooper, 2015)

Peter is a 12 year old boy. Since his parents divorced two years ago he now lives with his mother and her new husband, his 14 year old sister and his two older stepsisters. He was diagnosed with ADHD at the age of 8. Prior to Peter’s diagnosis he found it hard to concentrate in school and was restless and disruptive in class. At home he also displayed challenging behavior and had sleep issues. Currently he is being treated with medication and his family has received instructions on how best to support him by introducing structured daily routines. His sleeping issues are being treated with a special weighted blanket and a sleep hormone with good effect. Peter’s medical treatment is followed up on a regular basis by a specialist. He attends the local mainstream primary school. Since starting treatment Peter’s concentration in school has increased but he still struggles with his school work. In order to minimise Peter’s specific academic challenges, he has been given his own workstation in the classroom and his teachers have been advised to structure his school day. In addition, his teacher has been asked to ensure that before starting a new task Peter is listening and understands the task. These accommodations should assist Peter’s concentration and enable him to better complete his school work.

Peter’s ADHD is a lifelong condition, but with the right treatment his difficulties can be minimised. His future care is predicted to consist of mental health care provided by a range of specialists (doctors and nurses to follow up medication), social care and academic interventions and accommodations.

**Case scenario – Autism**

Autism or Autism Spectrum Disorder (ASD) is a set of pervasive developmental disorders, emphasised by the early onset of a triad of features: impairments in social interaction; impairments in communication; and restricted, repetitive, and stereotyped behaviour, interests, and activities (Lai, Lombardo & Baron-Cohen, 2013; World Health Organisation, 2016).

Mark is 2 years and 5 months old and has been newly diagnosed with autism. His development was considered normal up to the age of 15 months. He communicated with babble, held eye contact and reached motor skill milestones as expected. At 18 months Mark’s development began to stagnate, he experienced language regression and had difficulty maintaining eye contact. His parents described him as socially withdrawn, lacking interest in interacting with peers and having mannerisms with his hands. He also has a tendency to run askew, with his hands turned backwards. Currently, at diagnosis, Mark has been assessed as having severe and pervasive difficulties in all areas of the autistic spectrum. He is not developing age appropriate expressive or receptive language. In addition, his ability for social interaction as well as functional and symbolic play is found to be deviant and not age appropriate. This is considered stereotypical of autism. Mark’s gross and fine motor skills are also affected.

His future health and social care should be delivered in an environment where specialists with expertise in respect to autism can provide a care plan to support his development. It is also advised that his parents receive guidance in meeting Mark’s needs in the family setting. Mark’s condition is irreversible and he is expected to receive lifelong care and treatment, aimed at preventing further regression and develop new skills. The care will be provided by a multi-disciplinary team consisting of psychiatrists, psychologists, physiotherapists, occupational therapists, special community child and youth workers, social care workers, home care nurses and respite care services.

There is a high degree of heterogeneity across the Autistic Spectrum. Some children with autism will experience relatively little impairment and may not require the same level of expert care as Mark. It is expected, however, that the majority of children with autism, will require substantive care for their enduring complex care needs.

**SECTION 1: SYSTEMS OF CARE FOR CHILDREN WITH COMPLEX HEALTH NEEDS**

This section of the survey includes questions on the care of a child with ADHD and/or Autism in your country, adapted from the *Standards for Systems of Care for Children and Youth with Special Health Care Needs* (AMCHP and Lucile Packard Foundation, 2014). Please complete each section guided by the scenario provided of Peter and Mark and his family.

To ensure alignment in translation across the EU countries, some of the questionnaire keywords are specified here:

**Process:** A set of interrelated activities, which transform inputs into outputs.

**Procedure/guideline**: Description of actions done in a certain way.

**Policy:** A plan or course of action of decision makers at macro and meso-level to influence and determine decisions, actions, and other matters. This refers to a legal framework.

**A system:** A set of methods, procedures, arrangements, and activities that are carried out to form a unity or to meet a common goal.

This survey will, among other issues, deal with the integration of health and social care services. The organisation and integration of these services will vary substantially between countries. If there is a framework in your country for the integration of health and social services, please comment in the open-ended questions, as to whether the health and social needs of children with ADHD and Autism are adequately integrated or fragmented.

| SCREENING, ASSESSMENT, AND REFERRAL | | | | | |
| --- | --- | --- | --- | --- | --- |
| In my ~~country,~~ state there are policies and/or procedure(s) in place to assure that a child like Peter (ADHD) or Mark (Autism) receives *ongoing* preventative care screening and developmental checks with regard to their diagnosis. | | Policies  Yes  No  Yes  No | *If yes,* please provide reference(s) for the policy or procedures. | If yes, who starts this process? | |
|  |  | Procedure(s)  Yes  No  Yes  No | *If yes,* please provide reference(s) for the policies/ procedure(s) | If yes, who starts this process? | |
| *If there are policies and/or procedure(s) in place to ensure that a child like Peter* (ADHD) or Mark (Autism)  *receives ongoing preventative care screening and developmental checks with regard to their ADHD or Autism diagnosis*   1. please list the type of examinations provided | | ADHD  Autism | | | |
| 1. please name the healthcare providers that conduct these examinations (e.g. examination that screens for mental health comorbidity) | | ADHD  Autism | | | |
| 1. please name the social care providers that conduct these examinations (e.g. social well-being screening) | | ADHD  Autism | | | |
| In my ~~country~~,state there are policies and/or procedure(s) in place to document and communicate the results of such screening to all care services  (e.g. general practitioners/ primary care physicians/ paediatrician, community nurses, school, secondary care) caring for the child? ADHD AUTISM | | Policies  Yes  No  Yes  No | *If yes,* please provide reference(s) for the policy or policies. | *If yes,* who is in charge of the  communication process? | |
|  |  | Procedure(s)  Yes  No  Yes  No | *If yes,* please provide reference(s) for the procedure(s) | *If yes,* who is in charge of the  communication process? | |
| 1. the child’s parent(s) / guardian(s)? | | Policies  Yes  No  Yes  No | *If yes,* please provide reference(s) for the policy or policies. | *If yes,* who is in charge of the  communication process? | |
|  |  | Procedure(s)  Yes  No  Yes  No | *If yes,* please provide reference(s) for the procedure(s) | *If yes,* who is in charge of the  communication process? | |
| In my ~~country~~, state there are policies and/or procedure(s) in place to assess family capacities (e.g. knowledge and ability) that may influence providing care for the child with ADHD or Autism. | | Yes  No  Yes  No | If yes, please provide reference(s) for the policy or policies | If yes, please list who is in charge of  this assessment | |
| ~~Have you any further comments in relation to the screening, assessment, and /or referral of children with ADHD or Autism in your country state?~~ Is there anything else you think it would be valuable for us to know about screening, assessment and /or referral? | | ADHD  Autism | | | |
| ACCESS TO CARE | | | | | |
| There is a system in place in my ~~country~~ state to identify   1. all of the healthcare providers who care for children with ADHD or Autism. | ADHD  Yes  No  Autism  Yes  No | | *If yes,* who is in charge of this? | | |
| 1. all of the social care providers who care for children with ADHD or Autism | Yes  No  Yes  No | | *If yes,* who is in charge of this? | | |
| In my ~~country~~ state there is an agreed multi-disciplinary guideline of care for children with ADHD or Autism | ADHD  Yes  No  Autism  Yes  No | | *If yes,* who is in charge of this? | | |
| In my ~~country~~ state formal training on how to best care for children with ADHD or Autism is provided to the parent(s) / guardian(s). | ADHD  Yes  No  Autism  Yes  No | | *If yes,* which type of care are the parent(s) / guardian(s)  to provide? | | |
| *If parent(s) / guardian(s) receive formal training with regard to the care of children with ADHD* or *Autism*  are there policies and/or procedures in place to ensure that they have the capacity (e.g. knowledge and ability) to incorporate this into their child’s daily routine | Policies  ADHD  Yes  No  Autism  Yes  No | | *If yes,* please provide reference(s) for the policy or policies. | | |
|  | Procedure(s)  ADHD  Yes  No  Autism  Yes  No | | *If yes*, please provide reference(s) for the procedure(s) | | |
| Please provide a list of the statutory primary and secondary healthcare providers caring for children with ADHD or Autism in your ~~country~~ state (For example Child and Adolescent Psychiatry or paediatric units). | ADHD  AUTISM | | | | |
| Please provide a list of the voluntary healthcare providers caring for children with ADHD or Autism in your ~~country~~ state (For example respite services etc.) ^[[1]](#footnote-1)^. | ADHD  AUTISM | | | | |
| Please list the professions responsible, in your ~~country,~~ state for providing general healthcare services to children with ADHD or Autism in the community after treatment for an acute behavioural episode (For example general practitioner). | ADHD  AUTISM | | | | |
| Please list the professions responsible, in your ~~country,~~ state for providing social care services to children with ADHD or Autism in the community after treatment for an acute behavioural episode (For example specialised social care workers). | ADHD  AUTISM | | | | |
| Are there policies and/or procedure(s) in your ~~country,~~state which provide children like Peter or Mark and his family an overview of the interface between the health care and social care system responsible for Peter or Mark’s primary and secondary care? | Procedure(s)  ADHD  Yes  No  Autism  Yes  No | | | *If yes,* please provide reference(s) for  the procedure(s) | |
| *In my state…* |  | | |  | |
| … there are procedures in place to ensure that children like Peter and Mark are treated by the same healthcare provider / team of healthcare providers every time. | ADHD  Yes  No  Autism  Yes  No | | | *If yes,* please provide reference(s) for  the procedure(s) | |
| … there are procedures in place to ensure that children with ADHD/Autism are treated by the same social care provider / team of social care providers every time. | ADHD  Yes  No  Autism  Yes  No | | | *If yes,* please provide reference(s) for  the procedure(s) | |
| *In my state* |  | | |  | |
| … a child with ADHD/autism can access primary care regardless of care provided in secondary care and vice versa. | ADHD  Yes  No  Autism  Yes  No | | | *If yes,* please provide reference(s)  that supports your answer | |
| … there are policies and / or procedures in place, which *facilitate* or *hinder* children with ADHD/autism in accessing primary and secondary care simultaneously. | ADHD  Yes  No  Autism  Yes  No | | | Facilitators | Barriers |
| … there is transportation to care service for children with ADHD/Autism (e.g. access to special school, health assessment, etc.) in my ~~country~~ state is provided by | 1. the child’s parent(s) / guardian(s) with support from the state | | | Yes Yes  No No | |
|  | 1. the child’s parent(s) / guardian(s) without support from the state | | | Yes Yes  No No | |
|  | 1. the healthcare provider | | | Yes Yes  No No | |
|  | 1. the social care provider | | | Yes Yes  No No | |
| … there are policies and / procedure(s) in place to ensure that all information provided to families of children with ADHD or Autism is linguistically appropriate. | Policies  ADHD  Yes  No  Autism  Yes  No | | *If yes,* please provide reference(s) for the policy or policies. | | |
|  | Procedure(s)  ADHD  Yes  No  Autism  Yes  No | | *If yes*, please provide reference(s) for the procedure(s) | | |
| In my ~~country,~~ state there are policies and / procedure(s) in place to assure that all information provided to families of children with ADHD or Autism is culturally appropriate. | Policies  ADHD  Yes  No  Autism  Yes  No | | *If yes,* please provide reference(s) for the policy or policies | | |
|  | Procedure(s)  ADHD  Yes  No  Autism  Yes  No | | *If yes*, please provide reference(s) for the procedure(s) | | |
| Is there anything else you think it would be valuable for us to know about accessibility of care? | ADHD  Autism | | | | |
| CARE COORDINATION | | | | | |
| *In my ~~country,~~state* | |  |  | | |
| …there are policies and/ or procedures promoting access to care coordination for the child diagnosed with ADHD/Autism. | | Policies  ADHD  Yes  No  Autism  Yes  No | *If yes,* please provide reference(s) for the policy or policies | | |
|  |  | Procedure(s)  ADHD  Yes  No  Autism  Yes  No | *If yes,* please provide reference(s) for the procedure  or procedures | | |
| … there is a specific care pathway for children with ADHD or Autism | | Yes  Partly  No | *If yes,* please provide reference(s) to support your answer | | |
| *If there is a care pathway for children with ADHD and/or Autism are there policies or procedures that clearly describe each provider’s role in a care pathway for children with ADHD or Autism* | | ADHD Autism  Yes Yes  No No | | | |
| … there are procedures in place for ensuring a written personalised plan of care for children diagnosed with ADHD or Autism. | | ADHD  Yes  No  Autism  Yes  No | *If yes,* please list who is responsible for this? | | |
| … there is specific integration procedure and/ or policy of pathways for children with ADHD or Autism which combines primary and secondary care | | ADHD  Yes  No  Autism  Yes  No | *If yes,* please provide reference(s) for the procedure(s) | | |
| *If there is specific integration procedure and/ or policy of pathways for children with ADHD and Autism, which combine primary and secondary care, does this facilitate continuity?* | | ADHD  Yes  No  Autism  Yes  No | *If yes,* how? If possible please link to the evidence that  support your answer | | |
| …the written personalised plan of care for a child with ADHD or Autism is developed in consultation with   1. the child’s parent(s) / guardian(s) | | ADHD  Yes  No  Autism  Yes  No | *If yes,* who starts it? | | |
|  |  |  | *If yes,* who is in charge? | | |
| 1. Other healthcare professionals | | ADHD  Yes  No  Autism  Yes  No | *If yes,* who starts it? | | |
|  |  |  | *If yes,* who is in charge? | | |
| 1. Social care professionals | | ADHD  Yes  No  Autism  Yes  No | *If yes,* who starts it? | | |
|  |  |  | *If yes,* who is in charge? | | |
| …the personalised written care plan for a child like Peter or Mark integrates the following ;   1. developmental assessments | | ADHD Autism  Yes Yes  No No | | | |
| 1. mental health comorbidity assessments | | Yes Yes  No No | | | |
| 1. physical health | | Yes Yes  No No | | | |
| 1. oral health | | Yes Yes  No No | | | |
| 1. vision health | | Yes Yes  No No | | | |
| 1. hearing assessments | | Yes Yes  No No | | | |
| 1. social health | | Yes Yes  No No | | | |
| … hospital(s) that provide mental health care for children such as Peter or Mark (both outpatient and inpatient) have a discharge planning coordinator responsible for organising the transition of a child with ADHD or Autism from the hospital setting to the child’s home or another community based setting. | | ADHD  Yes  No  Autism  Yes  No | | | |
| ADHD and Autism are often associated with a high degree of comorbidity. Can you provide information on how children with ADHD and Autism are cared for in your ~~country~~ state in case of comorbidity? | | ADHD  AUTISM | | | |
| Is there anything else you think it would be valuable for us to know about coordination of care for children with ADHD or Autism in general your ~~country~~ state? | | ADHD  AUTISM | | | |
| COMMUNITY-BASED SERVICES AND SUPPORT | | | | | |
| *In my ~~country,~~state* | |  |  | | |
| … community-based services (e.g. local initiatives or voluntary organisations) are a key component of the care provided to children like Peter (ADHD) or Mark (Autism). | | ADHD  Yes  No  Autism  Yes  No | If yes, who coordinate the community-based care with the  hospital-based care | | |
| …family advocacy groups are involved in making recommendations to home and community-based services for children with ADHD or Autism. | | ADHD Autism  Yes Yes  No No | | | |
| … the parent(s)/guardian(s) and siblings of children with ADHD or Autism have access to professional psychosocial support. | | ADHD  Yes  No  Autism  Yes  No | *If yes,* who starts this process?  ADHD  AUTISM | | |
| …respite care is available for the parent(s) / guardian(s) children with ADHD and Autism | | Yes  No  Yes  No | ADHD  AUTISM | | |
| *If respite care is available, is it provided by* | | **State**  Yes  No  **Voluntary**  Yes  No  **Private sector**  Yes  No | *Please list the main provider for respite care in your country*  ADHD  AUTISM | | |
| … children with ADHD or Autism are offered individually tailored educational and / or training interventions or accommodations in response to their specific requirements. | | Yes  No  Yes  No |  | | |
| Is there anything else you think it would be valuable for us to know about the community based services and supports for children diagnosed with ADHD or Autism in your ~~country~~ state? | | ADHD  AUTISM | | | |
| FAMILY PROFESSIONAL PARTNERSHIP | | | | | |
| *In my ~~country~~ state parent(s) / guardian(s) of children with* ADHD or Autism  *are* | |  |  | | |
| … invited to participate in the development of policies and procedures affecting their children. | | ADHD  Yes  No | Autism  Yes  No | | |
| … included in national quality improvement initiatives for ADHD/autism. | | Yes  No | Yes  No | | |
| … involved in the review of patient and family information material on ADHD/autism for the public, to ensure cultural and/or linguistic competency. | | Yes  No | Yes  No | | |
| Anything else that it would be valuable for us to know about family professional partnerships relating to the care of children with ADHD or Autism in your ~~country~~ state? | |  | ADHD  Autism | | |
| TRANSITION INTO ADULTHOOD | | | | | |
| In my ~~country~~, state there are policies and procedure(s) in place to ensure continuity of care for adolescents with ADHD or Autism transitioning to adult specialists and community supports. | | ADHD  Yes  No  Autism  Yes  No | *If yes,* please provide reference(s) for the policies or procedures. | | |
| Please list the any barriers to continuity of care for adolescents with ADHD or Autism transitioning to adult care services in your ~~country~~.state | | ADHD  AUTISM | | | |
| Anything else it would be valuable for us to know in relation to the transition of adolescent with ADHD or Autism to adult care services? | | ADHD  AUTISM | | | |
| QUALITY ASSURANCE | | | | | |
| *In my ~~country~~,state* | |  |  | | |
| …there are quality assurance policies and/or procedures for service providers caring for children with ADHD or Autism. | | Policies  ADHD  Yes  No  Autism  Yes  No | *If yes,* please provide reference(s) for the policy or policies. | | |
|  |  | Procedure(s)  ADHD  Yes  No  Autism  Yes  No | *If yes,* please provide reference(s) for the procedure or procedures | | |
| …data is collected on the experience of care for children with ADHD or Autism from the perspective of   1. the parent(s) / guardian(s) | | Yes  No  Yes  No | *If yes*, who undertakes the collection and analysis of these data? ADHD  AUTISM | | |
| 1. the siblings | | Yes  No  Yes  No | *If yes,* who undertake the collection and analysis of these data? ADHD  AUTISM | | |
| 1. primary health and social care professionals | | Yes  No  Yes  No | *If yes,* who undertake the collection and analysis of these data? ADHD  AUTISM | | |
| Anything else it would be valuable for us to know about ensuring that children with ADHD or Autism in your ~~country~~ state receive quality care? | |  | ADHD  AUTISM | | |

**SECTION 2: EUROPEAN SURVEY OF CHANGE**

This section of the survey aims to provide a ‘barometer’ of the current situation of integration of care for children with complex care needs. You are asked to answer a number of questions to help us gain an understanding of current issues and debates on the issues of complex care for children with ADHD and Autism in your ~~country~~ state.

Please answer ***all*** the questions as accurately as possible and make use of the space for comments to clarify your information and to describe distinct characteristics of services/health organisations in your country.

1. **QUESTIONS ON THE CURRENT STATE OF DEVELOPMENT OF SERVICES FOR CHILDREN DIAGNOSED WITH ADHD and Autism**
   1. Please list the three most significant changes that have taken place in the integration of care services for children diagnosed with ADHD or Autism in your ~~country~~ state in the last five years?

ADHD

(i)

(ii)

(iii)

Autism

i)

(ii)

(iii

- 1. How would you evaluate the overall progress in the integration of care services for children with ADHD in your ~~country~~ state in the last five years? Please indicate with an ‘x’ the one statement that best describes this progress from the three options given below. Since January 2010 the integration of care for children with ADHD or Autism.

ADHD Autism

- - 1. has improved
    2. has remained very much the same
    3. has got worse
  1. Please list in order of importance the three main ***barriers*** to the integration of care services for children with ADHD and Autism in your ~~country~~ state at the present time:

ADHD Autism

(i)

(ii)

(iii)

- 1. Please list in order of importance the three main ***opportunities*** to the integration of care services for children with ADHD or Autism in your ~~country~~ state at the present time:

ADHD Autism

(i)

(ii)

(iii)

1. **QUESTIONS ON POLICY AND SOCIO-CULTURAL ISSUES**
   1. What strategies have been used to improve political awareness and government recognition of the need for integration of care services for children with ADHD and Autism in your ~~country~~ state in the last five years?

ADHD

Autism

- 1. Have there been any special funding initiatives for this group by government, private/voluntary care organisations, Non-Governmental Organisations, Organizzazione Non lucrativa di Utilità Sociale (ONUS),or donors in the last five years?

ADHD

Autism

- 1. ~~Has there been any involvement between your country and the European Union in relation to initiatives on managing the integration of care of children with ADHD in the last five years?~~

N/A

- 1. Has there been any major public discussion, debate or controversy about the needs of children with ADHD in your ~~country~~ state in the last five years?

ADHD

- 1. Has there been any major public discussion, debate or controversy about the needs of children with Autism in your ~~country~~ state in the last five years?

Autism

----------------------------------------------------------------------------------------------------------------------------------

Additional Questions

Thinking about mental health more broadly,

1. In an ideal world how would you like to see mental health services offered?
2. How do you think that fits with what is happening now?
3. What could we do now to take it closer to the ideal?
4. Do you think there is a difference between rural and urban areas in terms of the questions that have been asked, i.e. at a policy and procedural level

1. This list may not be exhaustive [↑](#footnote-ref-1)
